# Career adaptability and correlating factors among secondary vocational nursing students in China

**DOI:** 10.3389/fpubh.2024.1377323

**Published:** 2024-09-18

**Authors:** Yuhe Xiang, Lin Li, Qin Yang, YiQian Fang, Wenbin Xu, Xing Ding, Qian Yang

**Affiliations:** ^1^School of Nursing, Chengdu Medical College, Chengdu, China; ^2^Sichuan Cancer Hospital & Institute, Chengdu, China

**Keywords:** secondary vocational nursing students, career adaptability, locus of control, meaning in life, factors

## Abstract

**Background:**

Career adaptability significantly affects college students’ career happiness in the future, and it is essential to make a detailed evaluation of its correlation for making a targeted intervention plan. However, the applicability of occupational adaptability to secondary vocational nursing students is still an unexplored field in academic research.

**Objective:**

This study aimed to investigate the current situation of career adaptability and its influencing factors on secondary vocational nursing students in medical schools.

**Methods:**

A total of 1,414 secondary vocational nursing students from three secondary colleges and universities in Southwest China from July 2022 to September 2022 were selected for the survey. A questionnaire was used to assess secondary vocational nursing students’ sociodemographic, Career Adaptability, Psychological Control Sources, and the Meaning in Life. Statistical analyses were performed using Pearson’s correlation analysis, t-test, analysis of variance and multiple.

**Results:**

Secondary vocational nursing students scored (51.03 ± 9.64) for the meaning in life, (81.46 ± 25.39) for psychological control sources, and (94.12 ± 15.55) for career adaptability. Career resilience was significantly and positively correlated with the opportunity and internal control factors of psychological control sources (*r* = 0.091, −0.488, *p* < 0.01); and career adaptability was significantly and positively correlated with the sense of seeking meaning and possessing meaning factors of sense of meaning in life (*r* = 0.725, 0.734, *p* < 0.01); Multiple linear regression analysis revealed that academic performance, mother’s educational level, search for meaning, sense of meaningfulness, opportunities, and internal control entered the regression equation (*p* < 0.05), explaining 64% of the total variance.

**Conclusion:**

The overall career adaptability of secondary vocational nursing students is at a moderately high level, with significant individual differences closely correlated with locus of control, meaning in life, and career adaptability. Nursing educators can provide targeted intervention measures based on influencing factors to promote the development of secondary vocational nursing students’ career adaptability, enabling them to better adapt to future clinical work.

## 1 Introduction

Human resources for nursing are an important part of human resources for health, and the International Council of Nurses (ICN) has published a report highlighting the dire situation of human resources for nursing globally (1), with a global nursing workforce shortfall of up to 4.6 million by 2030 if no action is taken. In recent years, the construction of nursing human resources in China has been effective, and the nurse team has been greatly expanded (2). However, due to the aging of the population, the increase in the number of sub health people, and the reform of the medical system, the demand for nursing personnel has increased even more dramatically, and the “Outline of the Healthy China 2030” Plan proposes to reach the goal of 4.7 registered nurses per 1,000 permanent residents by 2030, with a shortfall of about 3 million nurses, and the gap in the number of nurses is still very serious (3). At present, China’s nurses still account for most of the college and junior college-educated personnel, although the education of registered nurses is constantly upgraded, secondary vocational nursing education is still an indispensable part of nursing education, coupled with the fact that in recent years, China has increased the support for vocational education (4, 5), so that vocational education is getting more and more attention from the society. Secondary vocational nursing students as an important reserve for clinical nursing work, but due to being in adolescence, coupled with the double pressure of facing academic and employment (6), they are more prone to psychological distress and behavioral problems, which leads to difficulties in successfully stepping into the clinical workplace and aggravates the nursing human resource shortage phenomenon. Therefore, nurturing secondary vocational nursing students’ capacity to confront career prospects and aiding their adaptation to the transition from learning to practical work is imperative.

Career adaptability plays an important role in facilitating nursing students’ adjustment to clinical work and refers to a socio-psychological resource that requires self-regulation in the face of different work tasks and role transitions (7). It develops along four dimensions: career concern, career control, career curiosity, and career confidence. Each dimension poses a core question that individuals must answer: “Do I have a future?,” “Who controls my future?,” “What do I want for my future?,” and “Can I achieve it?.” Individuals’ career adaptability evolves across these dimensions, ultimately shaping their unique attitudes, beliefs, and skills related to career planning, decision-making, and adaptation. Career concern assists in setting future goals, career curiosity accelerates exploration of potential selves and careers, career control grants individuals the right to choose their future, and career confidence enables individuals to construct an ideal future and overcome obstacles.

Career adaptability theory states that the goal of individual career development is to adapt to changing career environments, the core of which is the development of career adaptability (8). As a set of important personal resources, individuals can apply career resilience to cope with the changes and challenges of their careers, thus adapting to the career environment. Career adaptability has been shown to be positively associated with job satisfaction, career identity and employability (9, 10). Nursing students are an important part of the nursing population and have a significant impact on the construction of the nursing workforce and the development of hospitals. Nursing students are in the transition period from students to nurses, and the development of career resilience has an important role to play in promoting their career transition and professional growth. Pajic et al. (11) showed that a high level of career resilience motivates nursing students to engage in career planning and proactive development of skills, which improves their employability and role efficacy. Zhang et al. (12) found that nursing students with high levels of career adaptability were more successful in mastering career transitions, had higher employment rates, were able to make better career decisions and explorations, and were efficient in achieving career planning goals. Thus, good career adaptability can improve students’ career competence and help them successfully complete the transition from student to nursing worker role (13).

Previous studies have shown that the development of career adaptability is related to various factors, including age, academic performance, family role, etc. (14, 15). For example, the survey results of Liang et al. (16) show that the more support parents give, the higher the individual’s adaptability and adaptability will be. At the same time, with the continuous development of positive psychology, scholars at home and abroad gradually attach importance to the study of the sense of meaning of life. The sense of meaning of life means that individuals think that their lives are meaningful, can clearly realize the goals and missions of life, and constantly enhance their understanding and understanding of the meaning and mission in life in the process of pursuing this goal (17). The theory of expansion and construction points out that positive emotional experience can expand people’s cognitive and action abilities; Enhance personal resources, such as physical strength, intellectual psychology and social coordination, and promote personal growth and development (18). As a positive internal psychological resource, the sense of meaning of life can help individuals understand the purpose and meaning in life, so as to better determine the corresponding tasks for their future career development and realize their self-worth. At the same time, the sense of meaning of life can improve the individual’s resilience, and when encountering setbacks, it will help individuals to cope with challenges from all aspects of life and better realize their life ideals (19, 20). The research shows that when nursing students have a stronger sense of meaning in life, their satisfaction with their own environment is higher and their subjective well-being is stronger. In the face of the new career environment, I tend to face the difficulties and challenges in my work with a positive and optimistic attitude, and can take targeted measures to solve the problems, and turn the obstacles into the driving force for career development and opportunities for exercise, showing a high level of career adaptability (21, 22). Velly Ndlovu and other reports show that nurses who have a high level of sense of life are more likely to manage the current and expected career transformation, better relieve the work pressure, be full of curiosity and confidence in the future career development, and actively explore the career direction and possibility (23, 24).

The locus of control refers to an individual’s extensive expectation or attribution of whether the outcome of an event is controlled by himself or by external factors, and it is a general belief that the outcome of an event depends on himself or on luck, opportunity and influential others (25). Previous studies have analyzed individual cognitive ability and found that the development of career adaptability varies with individual psychological cognition (26). The psychological control source of internal control is that individuals believe that the outcome of events is determined by themselves and controllable. Zhu Lili et al. (27) research shows that internal control can highlight the self-efficacy of nursing students and show more positive psychological emotions, thus promoting the cultivation and improvement of nursing students’ career adaptability. However, there is still a lack of research on the relationship among career adaptability, sense of meaning in life and locus of control at the same time. In addition, China’s research on career adaptability is mainly carried out in the higher education environment, and few studies pay attention to the cultivation of career adaptability of nursing students in secondary vocational schools.

It is worth noting that there are differences in characteristics between students in secondary vocational schools and graduates of higher education. They acquire more technical abilities (occupation-specific skills) than their personal resources, but in the long run, specific hard skills will not have their advantages (28). Especially in today’s increasingly fierce competition in the job market (29), the society’s demand for talents is constantly improving, and it is becoming more and more difficult to meet and exceed expectations. In addition to professional and technical ability, secondary vocational nurses should also cultivate certain career adaptability to face unpredictable career changes and problems. Therefore, the following assumptions are put forward: (1) There are significant differences in career adaptability of nursing students in secondary vocational schools in some variables (gender, academic performance and educational level of parents); (2) Career adaptability is positively correlated with locus of control; (3) Career adaptability is positively correlated with the sense of meaning in life.

To sum up, this study aims to investigate the current situation of vocational nursing students’ career adaptability, and explore its key factors, including personal factors, sense of life meaning and source of psychological control, in order to provide useful guidance and practice for vocational nursing students to improve their career adaptability, so as to stabilize the clinical nursing talent pool and reduce the loss of nursing staff.

## 2 Methods

### 2.1 Study design and participants

Nursing students from three secondary colleges and universities in Southwest China from July 2022 to September 2022 were selected as the respondents. Inclusion criteria: secondary nursing students enrolled in school; those who voluntarily participated in this study. Exclusion criteria: those who transferred to another school in the middle of the year; those who took a leave of absence from school.

The sample size was determined according to the Kendall criterion, which means that the sample size should be 5–10 times the number of independent variables (30). Since this study included a total of 14 variables such as age, gender, registered residence, and taking into account the 20% loss of interview rate during the research process, the minimum sample size was estimated to be 88. A total of 1,414 respondents were included in this study, which meets the minimum sample size requirement. The study was approved by the Ethics Committee of Chengdu Medical College (2021NO.9), and the investigating students gave informed consent.

### 2.2 Measurement tools

The self-rating questionnaire comprised a section on sociodemographic characteristics, the Career Adapt-Abilities Scale (CAAS), the Internality, Powerful Others and Chance Scale (IPC), and the Meaning in Life Questionnaire (MLQ). Participants’ sociodemographic data included gender, age, registered residence, parents’ education level, family economic status, whether they are only children, whether they are student leaders.

Secondary vocational nursing students’ CAAS was measured using the Career Adapt-Abilities Scale (CAAS), which was developed by Savickas and Porfeli (31). The CAAS was translated by Hou et al. (32) and has 24 items over four dimensions: “career concern,” “career curiosity,” “career control” and “career self-confidence.” This instrument is scored on a 5-point Likert scale, and the higher the total score, the stronger the career adaptability. This scale is more mature and widely used by scholars in China, in this study, Cronbach’s alpha for the CAAS was 0.980.

Secondary vocational nursing students’ locus of control sources were measured using the internality、powerful others and chance scale (IPC), which was developed by Levenson et al. (33). The IPC was translated by Wang, et al. The scale consists of three dimensions, “Internality “, “powerful others,” and “Chance Scale “, with a total of 24 entries. This instrument is scored on a 6-point Likert scale; the total score for IPC ranges from 0 to 48. The higher score represents the individual’s tendency to be a source of psychological control. In this study, Cronbach’s alpha for the IPC was 0.948.

Secondary vocational nursing students’ MLQ was measured using the Meaning in Life Questionnaire (MLQ), which was developed by Steger et al. (17). The MLQ was translated by Wang, et al. The scale is divided into two sub-questionnaires, the presence of meaning (MLQ-P) and the search for meaning (MLQ-S), with five items each. This instrument is scored on a 7-point Likert scale, the higher the score, the higher the individual’s sense of meaning in life. In this study, Cronbach’s alpha for the MLQ was 0.916.

### 2.3 Data collection

The study used a convenience sampling method to select nursing students from three secondary vocational colleges and universities in southwest China. The questionnaires were distributed electronically through the Questionnaire Star platform and completed anonymously, and the questionnaires were uniformly collected and organized by the investigators.

A total of 1,483 questionnaires were distributed in this study, and the collected questionnaires were reviewed by two researchers, 69 invalid questionnaires were excluded, and 1,414 valid questionnaires were collected, with an effective collection rate of 95.34%.

### 2.4 Data analyses

All analyses were performed using IBM SPSS 26.0 (IBM Corp., Armonk, NY, United States). Quantitative information that conformed to normal distribution was described by mean ± standard deviation ( $\bar{x\;}\pm s$ ), and qualitative information was described by the number of cases and composition ratio. The t-test and analysis of variance (ANOVA) were used to compare demographic differences in secondary vocational nursing students’ career adaptability. The association between career adaptability, psychological control sources, and meaning in life was measured using the Pearson correlation. Multiple stepwise linear regression analysis was applied to determine the influencing factors of secondary vocational nursing students’ career adaptability, a two-sided *p* < 0.05 was considered statistically significant.

## 3 Results

### 3.1 Basic situation of secondary vocational nursing students

A total of 1,414 secondary vocational nursing students included in this study, aged (16.48 ± 0.80), were included, of whom 1,239 (87.6%) were female and 175 (12.4%) were male; 1,225 (86.6%) were from rural areas and 189 (13.4%) were from cities and towns; 1,038 (73.4%) were from families with average economic status; 1,273 (90%) were non-only children; 1,007 (90%) were student cadres 400 (28.3%), non-student cadres 1,014 (71.7%); academic performance in school: poor or worse 123 (8.7%), average or better 1,254 (88.7%), excellent 37 (2.6%); mother’s literacy level: elementary school and below 603 (42.6%), junior high school 657 (46.5%), senior high school or junior college 132 (9.3%), 22 (1.6%) university and above; father’s literacy level: 450 (31.8%) elementary school and below, 757 (53.5%) junior high school, 182 (12.9%) high school or junior college, 25 (1.8%) university and above (see Table 1).

**Table 1:** Basic situation of secondary vocational nursing students (*N* = 1,414).

Variables	Type	*N*	Percentage
Gender	Male	175	12.4
	Female	1,239	87.6
Registered residence	Countryside	189	13.4
	City	1,225	86.6
Family economic status	Poor	350	24.8
	Fair	1,038	73.4
	Good	26	1.8
Whether they are single children	Yes	141	10.0
	No	1,273	90.0
Whether to serve as a student leader	Yes	400	28.3
	No	1,014	71.7
Academic performance in school	Worse	21	1.5
	Poor	102	7.2
	Average	998	70.6
	Better	256	18.1
	Excellent	37	2.6
Father’s educational level	Primary and lower	450	31.8
	Junior	757	53.5
	High school or junior college	182	12.9
	College and higher	25	1.8
Mother’s educational level	Primary and lower	603	42.6
	Junior	657	46.5
	High school or junior college	132	9.3
	College and higher	22	1.6

### 3.2 Career adaptabilities, locus of control sources and the meaning in life scores of secondary vocational nursing students

The mean total score of career adaptability of secondary vocational nursing students was 94.12 ± 15.55 (range: 24–120); the mean scores of career concern, career curiosity, career control, and career confidence were 23.31 ± 4.10, 23.48 ± 4.16, 23.49 ± 4.11, and 23.85 ± 4.09, which indicated that the level of career adaptability of secondary vocational nursing students was relatively high; The mean score of the total locus of control sources of secondary school nursing students was 81.46 ± 25.39 (range: 12–144), and the mean scores of the three dimensions of powerful others, opportunity, and internal control were 24.77 ± 10.57, 25.71 ± 9.96, and 30.97 ± 7.47, respectively, which indicated that most of the secondary school nursing students tended to prefer internal control sources of psychological control; The mean value of the total meaning of life score of secondary school nursing students was 51.03 ± 9.64 (range: 10–70), of which the mean scores of the 2 dimensions of having a sense of meaning and seeking a sense of meaning were 24.78 ± 4.90 and 26.25 ± 5.44, respectively, indicating that the secondary vocational nursing students had a high level of meaning in life. In addition, the kurtosis and skewness values (34) in this study were within the acceptable range (see Table 2).

**Table 2:** Descriptive analyses of career adaptabilities, psychological control sources, and the meaning in life.

Variables	Entry score ( $\bar{X\;}\pm S$ )	Total score ( $\bar{X\;}\pm S$ )	Skewness	Kurtosis
Career adaptability	3.92 ± 0.65	94.12 ± 15.55	−0.270	0.676
Career concerns	3.89 ± 0.68	23.31 ± 4.10	−0.194	0.374
Career curiosity	3.91 ± 0.69	23.48 ± 4.16	−0.308	0.616
Career control	3.92 ± 0.68	23.49 ± 4.11	−0.313	0.564
Career confidence	3.97 ± 0.68	23.85 ± 4.09	−0.414	0.757
Locus of control	3.39 ± 1.06	81.46 ± 25.39	0.404	0.095
Powerful others	3.09 ± 1.32	24.77 ± 10.57	0.165	−0.496
Chance	3.21 ± 1.24	25.71 ± 9.96	0.199	−0.351
Internality	3.87 ± 0.93	30.97 ± 7.47	−0.115	0.999
Meaning in life	5.09 ± 0.97	51.03 ± 9.64	−0.128	0.314
Presence of meaning	4.93 ± 0.96	24.78 ± 4.90	0.053	0.331
Search for meaning	5.25 ± 1.09	26.25 ± 5.44	−0.355	0.483

### 3.3 Career adaptability scores of secondary vocational nursing students with different characteristics

The results of the univariate analysis showed that there was no statistically significant difference between gender, age, family economic status, being a lone child, and being a student leader in comparison with the total score of career resilience of secondary vocational nursing students (*p* > 0.05), and items with statistically significant differences are shown in Table 3.

**Table 3:** Comparison of career adaptabilities among secondary vocational nursing students with different socio-demographic characteristics (*N* = 1,414).

Socio-demographic characteristics	*N*(%)	Career adaptability score	*t*/*F*	*p*
Registered residence			−3.321	0.001
Countryside	1,225(86.6)	93.59 ± 15.25		
City	189(13.4)	97.61 ± 16.98		
Academic performance in school			15.622	<0.01
Worse	21(1.2)	82.00 ± 13.55		
Poor	102(7.2)	88.11 ± 18.31		
Average	998(70.6)	93.70 ± 15.09		
Better	256(18.1)	97.59 ± 14.70		
Excellent	37(2.6)	105.11 ± 14.38		
Family economic situation			8.871	<0.01
Poor	350(24.8)	92.46 ± 16.61		
Average	1,038(73.4)	94.41 ± 15.07		
Better	26(1.8)	105.19 ± 14.93		
Father’s educational background			2.965	0.031
Primary and lower	450(31.8)	94.00 ± 15.85		
Junior	757(53.5)	93.55 ± 15.00		
High school or junior college	182(12.9)	95.82 ± 16.58		
College and higher	25(1.8)	101.48 ± 16.31		
Mother’s educational background			3.267	0.021
Primary and lower	603(42.6)	93.38 ± 15.78		
Junior	657(46.5)	94.04 ± 15.08		
High school or junior college	132(9.3)	96.71 ± 16.00		
College and higher	22(1.2)	101.32 ± 17.34		

### 3.4 Correlation of career adaptability with locus of control sources and the meaning in life among secondary vocational nursing students

The results of the correlation analysis showed that locus of control sources and career adaptability were statistically significant (*r* = 0.199, *p* < 0.01). Among all dimensions of locus of control sources, only internal control (*r* = 0.488, *p* < 0.01) and opportunity (*r* = 0.091, *p* < 0.01) were significantly correlated with career resilience. At the same time, the relationship between powerful others and career adaptability (*r* = 0.048, *p* > 0.05) was not statistically significant. Sense of meaning in life was statistically significant with career adaptabilities (*r* = 0.783, *p* < 0.01), where both sense of seeking meaning (*r* = 0.725, *p* < 0.01) and sense of having meaning (*r* = 0.734, *p* < 0.01) were positively correlated with career adaptabilities (see Table 4).

**Table 4:** Correlation analyses (*R* values) of career adaptabilities with internality powerful others and the meaning in life (*N* = 1,414).

Variables	1	2	3	4	5	6	7	8
1. Total score for career adaptability	1							
2. Total score for psychological control sources	0.199**	1						
3. Internality	0.488**	0.783**	1					
4. Powerful others	0.048	0.948**	0.588**	1				
5. Chance	0.091**	0.956**	0.623**	0.914**	1			
6. Total score for meaning in Life	0.783**	0.242**	0.512**	0.093**	0.135**	1		
7. Presence of meaning	0.725**	0.304**	0.504**	0.170**	0.216**	0.939**	1	
8. Search for meaning	0.734**	0.140**	0.448**	−0.005	0.026	0.924**	0.735**	1

**p < 0.01.

### 3.5 Factors associated with career adaptabilities

The total score of career adaptability of secondary vocational nursing students was used as the dependent variable, and nine variables with statistically significant differences in one-way and correlation analyses (registered residence, school academic performance, family economic status, father’s educational background, mother’s educational background, internal control, opportunity, sense of meaning-seeking, and sense of meaning-holding) were used as the independent variables in the multivariate regression analyses and the variable assignments in the multiple linear regression are shown in Table 5.

**Table 5:** Assigning values to variables.

Variables	Assigning values
Registered residence	Countryside = 0; City = 1
Academic performance in school	Worse = 0; Poor = 1; Average = 2; Better = 3; Excellent = 4
Family economic situation	Poor = 0; Average = 1; Better = 2
Father’s educational background	Primary and lower = 0; Junior = 1; High school or junior college = 2; College and higher = 3
Mother’s educational background	Primary and lower = 0; Junior = 1; High school or junior college = 2; College and higher = 3

The results of multifactorial analysis showed that secondary vocational nursing students with average (*β* = 0.149, *p* = 0.015), good (*β* = 0.138, *p* = 0.010), or excellent (*β* = 0.059, *p* = 0.026) academic performance in school had higher levels of career resilience; secondary nursing students whose mothers had higher levels of education (*β* = 0.045, *p* = 0.017) had higher levels of career resilience; secondary nursing students whose source of psychological control favored internal control (*β* = 0.217, *p* < 0.001) had better; and secondary nursing students whose source of psychological control favored opportunity (*β* = −0.131, *p* < 0.001) had lower; secondary nursing students with a good sense of meaning-seeking (*β* = 0.377, *p* < 0.001) and a good sense of meaning-having (*β* = 0.352, *p* < 0.001) had higher levels of career resilience, explaining 64% of the total variance (see Table 6).

**Table 6:** The multiple stepwise linear regression analysis of career adaptability of secondary vocational nursing students (*N* = 1,414).

Variables	B	SE	Beta	*t*	*p*
(Constant)	24.400	2.495	–	9.781	<0.001
Academic performance in school					
Worse	–			–	
Poor	1.352	2.249	0.023	0.601	0.548
Average	5.096	2.087	0.149	2.442	0.015
Better	5.572	2.148	0.138	2.594	0.010
Excellent	5.788	2.600	0.059	2.226	0.026
Mother’s educational background					
Primary and lower	–			–	
Junior	1.062	0.578	0.034	1.838	0.066
High school or junior college	1.820	1.043	0.034	1.745	0.081
College and higher	5.610	2.340	0.045	2.397	0.017
Internality	0.451	0.052	0.217	8.740	<0.001
Chance	−0.204	0.035	−0.131	−5.853	<0.001
Presence of meaning	1.118	0.081	0.352	13.760	<0.001
Search for meaning	1.079	0.071	0.377	15.139	<0.001

*R2 = 0.644; calibration R2 = 0.640; F = 157.697; SE, standard error.*

## 4 Discussion

Well-developed career adaptability is a key factor for nursing students to achieve career success and can facilitate the smooth transition of nursing students from school to work roles. With the development of society and economy, career development has become more and more complex, especially in the context of the global nursing human resource shortage (35), and the increase in labor demand will bring many challenges to future students who graduate from the clinical work environment. Secondary vocational nursing students are an even more important reserve for professional nursing staff. Therefore, it is important to understand the current situation of career adaptability and the factors influencing it *among* secondary vocational nursing students to improve their intention to continue in the nursing profession and stabilize the nursing workforce.

### 4.1 Factors influencing career adaptability in secondary vocational nursing students

The results of this study showed that the total score of secondary nursing career adaptability was (94.12 ± 15.55), which was in the medium-high level, indicating that secondary nursing students have a certain degree of resilience to future career development, which is consistent with the findings of Shi (36). The reason for this may be that with the development of the nursing discipline in China, the training system for nurses is becoming more and more perfect, and at the same time, China has opened a variety of pathways for secondary vocational students at the policy level, which provides more possibilities for career development for secondary vocational nursing students. Among all the dimensions, the career self-confidence dimension scored the highest, which may be because secondary vocational nursing students are in adolescence, have a lot of unrealistic and beautiful fantasies about their future work, have high expectations and strong self-confidence, and believe that they can make a difference in their future clinical work, which stimulates their career self-confidence; and the lowest score of the career concern dimension may be related to the fact that most of the secondary vocational nursing students have not yet entered into the real clinical work environment, and the study found that (37) close contact with the career achievers in the real career environment can make them pay more attention to the development of their careers and their direction, which will improve their career concern. Therefore, nursing educators should plan and purposefully set up personalized career resilience education programs, and career planning guidance, and suggest more career courses centered on self-reflection (38) to do career-oriented exercises for secondary nurses. At the same time, the power of career role models can be used to stimulate and guide nursing students to pay attention to their careers, to comprehensively improve their career adaptability.

The results of multiple linear regression analysis in this study showed that academic performance in school and mother’s education were the influencing factors of career adaptability of secondary vocational nursing students. In this study, the career adaptability scores of secondary vocational nursing students with excellent academic performance and mothers’ education level of university and above were the highest, which was consistent with the findings of Jiang R et al. (39), which found that there was an interaction between career adaptability and academic performance and that secondary vocational nursing students with excellent academic performance tended to have a higher sense of self-efficacy, clearer goals, and more confidence in their future career development and would develop their abilities to achieve better career development. They will develop their abilities to achieve better career development and therefore have a higher level of career adaptability. In addition, mothers in Chinese families are more concerned about the development of their adolescent children (40), and the more educated mothers are, the more they pay attention to education and knowledge learning, and at the same time, they can give more support to make secondary nursing students have more resources (41). This also suggests that nursing educators should strengthen communication and cooperation with parents of secondary nursing students, guide parents to pay active attention to their children’s career development, mobilize their children’s enthusiasm for learning; and provide academic support as much as possible to stimulate their intrinsic potential, to improve the individual’s initiative for self and career exploration.

### 4.2 Effects of secondary vocational nursing students’ locus of control sources on career adaptability

Locus of control reflects an individual’s perception of behavioral outcomes, whether they are seen as being actively determined or passively influenced. Individuals with a higher internal locus of control tend to attribute success to their own efforts or attitudes, thereby fostering greater confidence in their own development (42). The results of this study showed that secondary vocational nursing students who tended to be internally controlled had higher levels of career adaptability (*p* < 0.001). Botha and Dahmann (43) found that individuals who tended to be internally controlled had greater self-control, and adolescents with greater self-control tended to use more effective strategies to pursue their personal goals, which resulted in higher levels of well-being and life satisfaction (44). Thus when internally controlled secondary vocational nursing students are faced with uncertain career choices or developments, they may be more inclined to take the initiative to confront problems, solve them, and adapt to changing career demands through positive changes in themselves.

It is salient to observe that, while this study denoted that the incongruity within the powerful others dimension and career adaptability lacked statistical significance, the dimension of opportunity as a source of locus of control manifested a significantly negative correlation with career adaptability.

This, to a certain extent, implies that a higher inclination towards external control as the locus of control among secondary vocational nursing students corresponds to a diminished level of career adaptability. The sub-survey shows that secondary vocational nursing students who tend to be externally controlled believe that opportunities will affect their success more and present a state of being at the mercy of their future career development without a clearer career plan, which is also consistent with the results of the study by Wang et al. (45). This suggests the importance of locus of control sources to career adaptability, locus of control sources are not static, educators should pay attention to the management of locus of control sources of secondary vocational nursing students during the school period, and it is recommended that teaching guidance be provided according to the characteristics of different sources of locus of control, guiding them to change to the internal control type, developing personal potential, and enhancing career adaptability (46). Cazan and Dmitrescu (47) found that positive thoughts were associated with higher internal control, which can help people choose behaviors that are consistent with their needs, interests, and values, and lead to greater confidence in their ability to have higher internal control. Therefore, nursing educators may give students certain positive thinking interventions and integrate positive thinking training into their teaching programs, which may be important for improving the psychological adaptability, maintaining a healthy state of mind, and good internal control of secondary vocational nursing students.

### 4.3 Effects of secondary vocational nursing students’ meaning in life on career adaptability

The results of this study showed that secondary vocational nursing students’ sense of the meaning in life plays an important role in the development of their career resilience, and the stronger their sense of the meaning in life, the higher the level of career adaptability which is consistent with the findings of To et al. (48). The theory of sense of meaning in life holds that seeking the purpose and meaning in life is the basic motivation for human existence, and the sense of meaning in life is the individual’s attitude and cognition towards the meaning and value of life, and the sense of meaning in life, as a positive factor, can continue to positively predict mental health, motivate individuals to achieve self-transcendence of the intrinsic motivation to help people adapt to the environment positively and effectively, and alleviate the individual’s anxious and depressive moods (49). Therefore, people with higher levels of meaning in life are abler to feel warmth, happiness, and fulfillment, all of which can help individuals better adapt to the dilemmas they face at work. In addition, it has been found that a sense of the meaning in life is significantly positively correlated with a sense of self-worth, life satisfaction, subjective well-being, and positive emotions, and when individuals have a stronger sense of the meaning in life, the more positive emotions they generate, and a good state of mind and sense of self-efficacy can help individuals cope with the problems they encounter in their career development, thus promoting the enhancement of the level of career adaptability (50, 51).

As the reserve manpower of the nursing profession, secondary vocational nursing students are in the critical period of personality shaping, and only when they correctly understand the significance of the existence of patients’ lives will they respect and honor life more, improve the level of career adaptability, and better devote themselves to clinical nursing work (52). Therefore, nursing educators can consider the following aspects in the process of improving the level of career adaptability of secondary vocational nursing students: firstly, nursing administrators and educators of the institutions should focus on the characteristics of secondary vocational nursing students, combine clinical teaching, consciously combine career education with life education, and carry out the comprehensive course of “life-career” to guide secondary vocational nursing students to form a correct view of life and values and maintain a positive attitude to work and life, to improve career adaptability. To improve career adaptability, they should consciously combine career education with life education and carry out the “life-career” comprehensive program to guide secondary vocational nursing students to form a correct outlook on life and values and to maintain a positive mindset to cope with work and life. Secondly, in addition to classroom education, colleges and universities should organize more recreational activities to enrich the spare time of secondary vocational nursing students, stimulate their enthusiasm for life through lectures, group counseling, psychological decompression, etc., and truly understand the meaning in life, help them to firmly establish their future career direction and enhance their confidence in facing the future, to further enhance the career adaptability of secondary vocational nursing students.

### 4.4 Limitations

This study also has certain shortcomings. First, the cross-sectional design was used, and the target population was limited to secondary vocational nursing students in Southwest China, so the sample size selection has some limitations, in the future, a multi-region, multi-center longitudinal survey can be conducted to explore the trajectory of career adaptability of secondary vocational nursing students at different stages of their careers. Secondly, the level of career adaptability of secondary vocational nursing students is influenced by various aspects; therefore, other predictors can continue to be explored in future studies.

## 5 Conclusion

In summary, the career adaptability of secondary vocational nursing students is in the middle to the upper level, meanwhile, this study found that academic performance in school, mother’s educational level, locus of control, and sense of the meaning in life affect the development of career adaptability of secondary vocational nursing students to a certain extent, which also highlights the weakness of the career education of secondary vocational nursing students and the key content of the career counseling that needs to be paid attention to. Vocational education needs to start at school and continue throughout the life cycle. It requires the inclusion of external social support systems, such as psychological counseling, parental peer support, and career guidance, and the establishment of internal and external linkage management mechanisms to guide secondary vocational nursing students towards an internal control shift, to strengthen their knowledge of the meaning in life, to enhance their positive career experience and exploration, and to improve the level of career adaptability.

## Data Availability

The raw data supporting the conclusions of this article will be made available by the authors, without undue reservation.
